# Perceived Risks, Mitigation Strategies, and Modifiability of Telehealth in Rural and Remote Emergency Departments: Qualitative Exploration Study

**DOI:** 10.2196/58851

**Published:** 2025-04-15

**Authors:** Christina Tsou, Justin Yeung, Melanie Goode, Josephine Mcdonnell, Aled Williams, Stephen Colin Andrew, Jenny Tetlow, Andrew Jamieson, Delia Hendrie, Christopher Reid, Sandra Thompson

**Affiliations:** 1 WA Country Health Service Curtin University Perth Australia; 2 WA Country Health Service Perth Australia; 3 Curtin University Perth Australia; 4 Western Australian Centre for Rural Health University of Western Australia Perth Australia

**Keywords:** emergency telemedicine, implementation effectiveness, clinical effectiveness, risk aversion, risk mitigation, rural and remote, emergency departments

## Abstract

**Background:**

Telehealth is a recognized and rapidly evolving domain in the delivery of emergency medicine. Research suggests a positive impact of telehealth in patients presenting for emergency care; however, the regional challenges of acute telemedicine delivery have not been studied. The WA Country Health Service (WACHS) established the Emergency Telehealth Service (ETS) in 2012 to provide telehealth and other technology-enabled services to regional Western Australian hospitals and clinics. The WACHS ETS supports 87 rural and remote WACHS-operated hospitals as well as 10 non-WACHS health clinics via high-definition audio-visual equipment installed in the resuscitation bay of the emergency department (ED) at each site. This 12-year practical application of emergency telemedicine offers a unique opportunity to explore the experiences and perceptions of clinicians delivering virtual care to rural and remote communities.

**Objective:**

This study explores the perceptions of ETS clinicians regarding acceptability, appropriateness, and clinical decision-making when delivering emergency telemedicine in rural and remote settings.

**Methods:**

This qualitative study used semistructured interviews to explore the perspectives of ETS clinicians regarding the factors influencing their clinical decision-making. It explored how ETS clinicians determine and modify clinical risks associated with using audio-visual equipment to deliver care. Emerging themes were compared with the concepts arising from the interim guidance of the Medical Board of Australia, and both the Australian and New Zealand, and American Colleges of Emergency Medicine.

**Results:**

Overall, 16 doctors, 4 clinical nurse coordinators, and a nurse educator from WACHS ETS provided their experiences and perspectives. Accurate clinical decisions, especially regarding patient disposition, were crucial to virtual care. Timeliness and accuracy were enhanced through a mutual learning model grounded in the local context. Mitigation strategies such as improvisation and flexible technology use compensated for technological barriers. Nonmodifiable risk factors included patients’ presenting complaints, clinical urgency of presentation, ED capability, clinician scope of practice, and, if a transfer was required, the distance between the ED of original presentation and the hospital of definitive care.

**Conclusions:**

Telehealth can enhance clinical decision-making in rural and remote EDs, and ETS clinicians can prioritize patient safety through a lens incorporating both local hospital capabilities and community contexts. Even for the most experienced clinicians, telehealth was not comparable to face-to-face communication in all circumstances. The impact of the ETS on the scope of the regional emergency medicine practice and on the building of clinical skills warrants further study in relation to its overall effectiveness and cost-effectiveness in rural and remote EDs. These findings identify areas for further qualitative research while providing a rich contextual background for rigorous quantitative analysis of the effectiveness of the ETS.

## Introduction

### Background

Telehealth has rapidly emerged as a means of providing health advice and care to people at sites where health providers at the hospitals of initial presentation (presenting hospitals) may lack capability or require assistance with the diagnosis and treatment of presenting clinical conditions. In recognition of the rapidly evolving domains of telehealth in the delivery of emergency medicine (EM) in the United States [1] and Australia and Aotearoa (New Zealand) [2], the Australasian College of Emergency Medicine (ACEM) [2], American College of Emergency Physicians [1], and Medical Board of Australia [3] deliberated on interim principles, considerations, and minimum standards to guide emergency telemedicine (“the interim guiding principles from the Colleges of Emergency Medicine”). Telehealth in the emergency department (ED) aspires to improve the quality of health care for patients in remote and other settings by increasing access to specialists and optimizing health service utilization and disposition coordination, including timely access to definitive care [2].

### Guiding Principles on Emergency Telemedicine Implementation

These interim principles provide minimum standards for telehealth practice in the ED. As the Colleges of Emergency Medicine have identified, the use of telehealth in EM is in the formative stage and currently lacks a strong evidence base for clinical practice in the context of emergency care.

The ACEM and Australian New Zealand Fellow of ACEM (FACEM) Telemedicine Community of Practice (ANZFTCOP), superseded by a formal ACEM Emergency Telehealth Network, raised the importance of local context and the unplanned and exigent nature of emergency care [3]. The ANZFTCOP distinguished the nature of emergency medical practice from the broader telehealth discussion as it involves the provision of acute care without an ongoing clinical relationship either in-person or virtually [3]. The Community of Practice also pointed to circumstances where it may not be possible to access emergency physician expertise without recourse to telemedicine [3]. This is common for many rural and remote Australian EDs. The ANZFTCOP suggested that the decision regarding the appropriateness of conducting a telemedicine session instead of an in-person consultation should be based on individual clinical circumstances and expert clinician judgment [3]. Table 1 provides an amalgamated summary of the key guiding principles from these position statements and guidance documents.

**Table 1:** Amalgamated summary of the interim guidance on emergency telemedicine implementation.

Key themes	What is emergency telehealth and what should it do?	What is not emergency telehealth and what should it not do?
Locally grounded	Should be an important complement for locally and regionally provided comprehensive health care. Should be part of regional models of emergency care delivery validated by all relevant stakeholders across the public health system. Should have regard for the local context and be grounded in the relationship with local clinicians including but not limited to knowledge of the local population, service availability, and patient pathways. Should have appropriate escalation procedures in place to activate emergency services.	Should not replace local care. Should not be considered as a substitute for face-to-face consultations. Should not involve low-value care that would not have otherwise been provided. Should not be a stand-alone solution to address health workforce capacity and maldistribution.
Of similar quality and standard to ordinary care	Assessments and consultations: (1) should be thorough within the limitations of telehealth and virtual consultations, (2) should replicate the components of an emergency medicine consultation, and (3) should be cognizant of the peculiarities or distinctive requirements of specific conditions. Should implement quality assurance programs to monitor clinical performance, patient outcomes, and integrated ongoing care.	—^a^
Timely care	Should ensure that definitive care is not delayed.	Should not create additional barriers to accessing emergency medical care.
Appropriateness of care	Should ensure that the patient’s presenting problem is suitable to be assessed and managed remotely.	Not appropriate for all clinical consultations.

^a^Not applicable.

### Study Setting

The WA Country Health Service (WACHS) is one of the largest country health services in the world in terms of geographical coverage (2.55 million square kilometers, 96% of the land mass of Western Australia), with a population of 531,934 (18% of the Western Australian population; population density of 0.21 per square kilometer). It is organized into 7 distinct and diverse regions with 118 health facilities, accounting for 41% of the total number of emergency presentations across Western Australia (over 40,000 presentations per year) [4]. Most hospitals outside the large regional centers are staffed mainly by nursing staff supported by a resident general practitioner (GP) credentialed to provide medical services at the hospital. There are variable numbers of consultant specialists providing services at the larger regional resource centers. The majority of WACHS hospitals do not have on-site access to emergency medicine physicians (FACEMs).

The WACHS established the Emergency Telehealth Service (ETS) in 2012 to provide telehealth and other technology-enabled services to regional Western Australian hospitals and clinics. The ETS is the earliest and most established service stream of the WACHS Command Centre, which supports 87 WACHS hospitals and 10 additional non-WACHS facilities through high-definition videoconferencing services [5]. The Command Centre is a 24/7 virtual clinical and operational hub including services for general medical inpatients, mental health, midwifery, after-hours palliative care, and transfer coordination. It supports clinicians in regional hospitals and nursing posts by providing ready access to specialist clinicians who use technology, videoconferencing, and real-time data to assist in delivering quality patient care [6].

In addition to over 38,000 clinical consultations per year, the ETS supports rural and remote clinicians to maintain skills and knowledge through an education program and opportunities for professional development [5]. The consulting clinicians are located across multiple geographical areas (Perth, across regional Western Australia, other Australian jurisdictions, and internationally). Most medical practitioners are FACEMs complemented by EM-credentialed GPs and emergency nurse practitioners. The central hub of the Command Centre is based in Perth, and referrals are coordinated by a team of clinical nurses and clinical nurse consultants.

Referrals to the ETS vary across hospital types but are mainly from small hospitals (81.5%), nursing posts (9.7%), and integrated district health services (8.2%), and the ETS is least used by regional resource centers (0.7%) [4,7]. The proportions of ED presentations involving the ETS also vary across hospital types, with nursing posts and small hospitals involving the ETS in 21.3% and 23% of all ED presentations, respectively. Between July 2018 and April 2023, the uptake for nursing posts ranged from 2.8% for nonurgent presentations to 62.1% for emergency presentations that must be attended within 10 minutes. For small hospitals, the uptake ranged from 5.3% for nonurgent presentations to 47.9% for presentations requiring resuscitation. The more remotely located EDs appear to have greater reliance on the ETS especially for the management of more urgent cases [4,7].

### Evidence on Telehealth Implementation in Rural and Remote EDs

Studies have identified factors that negatively influence the experiences of regional clinicians in rural and remote EM practices. These include [8,9] the lack or absence of medical backup or resources, the geographical and social isolation of rural communities, poor health system coordination, the loneliness experienced by regional clinicians, and the challenges of health care provision in geographically isolated locations [10-12].

The challenges these conditions pose are community specific [8], and studies found that rural and remote EM physicians drew on the strength of professional relationships to improvise, solve problems, and create systems of support to meet local needs in order to survive and thrive in the middle of rural and remote challenges [8,9].

Published studies on the use of telehealth in EM suggested positive impacts on patient care, including benefits such as cost reduction, improved quality of care, reduced mortality rate, reduced patient treatment time, and reduced time between first contact and treatment [13]. Moreover, in the rural and remote context, studies have reported reduced patient transfers from rural facilities to major centers [10-12,14-16] and improved capability of rural centers [10,17,18]. However, studies have not discussed the complexities of rural and remote EM practices in depth or taken them into account in their analyses.

The use of telehealth in rural and remote EDs to help alleviate these regional EM practice challenges has not been studied. Whether or how emergency telemedicine in rural and remote settings improves patient health outcomes and the factors influencing the clinical decisions of emergency telehealth clinicians remain uncertain. There is a need for quality evidence to better understand these factors to help identify the gap in the effectiveness of emergency telehealth [12,14,15,17,19-27].

Studies have, however, identified challenges in the implementation of virtual care, and emergency telehealth is one of the practice areas considered [14,22,23]. These include technical difficulties [11,12,23,25-27], factors impeding the process of care [10,14,16,19-21,24-26], and factors impacting patient privacy, confidentiality, and data security [24-26].

It has been proposed that to understand the factors influencing job satisfaction, the complex environments around rural and remote EM practices must be considered [8]. However, the impact of environmental factors on clinical decision-making in rural ED practice has not been considered.

The clinicians’ perceived clinical risks have been identified as a challenge in rural and remote EM practices [8,9]; however, how individuals perceive the risks in rural and remote emergency telehealth practices and the impacts on their clinical practices have not been explored previously. In rural and remote EDs, it is possible to assume that when the perceived clinical risk is high, the approach to diagnosis and treatment may change, leading to increased rates of transfer. The distinctions between these perceived clinical risks when delivering face-to-face versus virtual EM services warrant investigation.

To the best of our knowledge, specific links between the challenges of clinical reasoning and decision-making in virtual emergency care and the rural and remote context have not been identified. However, these factors are critical for understanding the effectiveness of implementing telehealth in rural and remote EDs.

### Study Aims

From the perspective of WACHS clinicians delivering the ETS, this study explored the perceptions of emergency telehealth clinicians regarding the acceptability, appropriateness, and determinants in clinical decision-making when delivering emergency telehealth in rural and remote settings. This study aims to understand the following: (1) the factors influencing clinical decisions during an emergency telehealth consultation; (2) the impact of involving emergency telehealth clinicians in rural and remote ED consultations on the health workforce and the safety of clinical service delivery; and (3) how the clinical risks associated with emergency telehealth consultation can be modified, including whether improving the status of factors that are amenable to change can positively influence the safe and effective delivery of the ETS.

## Methods

### Overview

This qualitative study used semistructured interviews to explore the perspectives of ETS clinicians regarding the factors influencing their clinical decision-making. It explored how ETS clinicians determine and modify associated clinical risks. Emerging themes were compared with the concepts arising from the interim guidance of the Medical Board of Australia and both the Australian and New Zealand, and American Colleges of Emergency Medicine. The WACHS ETS was used to examine contextual factors influencing the quality and perceived effectiveness of emergency telehealth in rural and remote areas.

### Sampling Techniques and Study Participants

A combination of convenience, snowball, and purposive sampling was used to obtain a diverse perspective on the ETS model of care. Convenience sampling was required given the nature of ED work and participant availability to recruit FACEMs, GPs, ETS nurse coordinators, and clinical nurses. A purposive sampling of WACHS ETS physicians who were interstates or overseas occurred toward the end of the participant recruitment.

On December 17, 2020, an invitation email was sent to all ETS doctors and nurses by one of the FACEM members of the research team. This was followed by individual follow-up emails on January 22, 2021, to ETS doctors, as well as emails and in-person follow-ups by one of the ETS clinical nurse consultants in the research team.

### Interview Guide Development

The interview protocol (guide) was developed in collaboration with the clinical researchers at the Command Centre. This interview guide was then distributed to the full research team for review.

After piloting the interview protocol with a select leadership group, the protocol was updated to include probes on whether an electronic stethoscope would help provide confidence in clinical decision-making, as well as adding questions related to the clinical governance, service legitimacy, and legality of the ETS practice. Minor changes were also made to the wording and ordering of the content areas and probes within each content area. The final version of the interview protocol is presented in Multimedia Appendix 1.

### Simultaneous Data Collection and Analysis

Semistructured in-depth interviews explored the experiences and opinions of the clinicians involved in ETS implementation and the factors considered in clinical decision-making.

Participants were given the option to participate in an interview via MS Teams (Microsoft Corp) or phone, from a place and at a time of their choosing. Interviews were audio recorded, automatically transcribed, and repeatedly played back in the open-coding phase of the analysis to validate the accuracy of the transcription. Due to the nature of emergency work, the recruitment and conduct of the interview occurred opportunistically to catch moments when the clinicians were available. This meant that interviews sometimes occurred outside rostered work times, with some conversations interrupted by work-related demands.

Data analysis proceeded simultaneously with data collection and issues arising from information [28]. The early phase of the analysis included open and axial coding [29], identifying the emerging themes with open coding from clinician interviews. The emerging themes were then compared with the guiding principles identified from the interim guidance documents of the Colleges of Emergency Medicine and Medical Board of Australia. The key guiding principles were introduced as axial codes to place the open codes within the context of EM practice. In the final phase of the analysis, risk factors considered by clinicians in their clinical decision-making during an emergency telehealth consultation were extracted and classified into technology related, presenting hospital/location related, or EM related. The modifiability of each of the factors was assigned by the lead author and confirmed by co-authors based on author experience, the rural and remote context of Western Australia, the public health service landscape, and the resources available to the WACHS. The factual findings reported in this paper were discussed with and confirmed by the WACHS Command Centre.

### Ethics Approval

This research obtained ethics approval from the WACHS Human Research Ethics Committee (approval number: RGS0000003076) and reciprocal ethics approval from the Curtin University Human Research Ethics Committee (approval number: HRE2019-0740-09). Site access was governed by the WACHS research governance policy and procedures. A participant information and consent form was attached to individual email invitations to the participants to review, sign, and return before the scheduled interview. For participants who attended the interview but were unable to sign the consent form, verbal consent was obtained before the commencement of the interview. Interview recordings were transcribed verbatim by the first author. The transcripts and recordings were allocated a participant code and stored in a password-protected electronic folder that is separated from the folder where the participant register is stored.

## Results

### Interviews and Participants

Most interviews were conducted via MS Teams (17/21, 81%), and the remaining were conducted by phone (4/21, 19%). Participants were in their private homes, a dedicated home office space, or Western Australian health facilities.

Sixteen WACHS ETS doctors and 5 ETS nurses participated in the interviews. There were 9 FACEMs, 5 GPs, 2 ED registrars, 4 clinical nurse coordinators, and 1 nurse educator. All participating ETS nurses were based at the Command Centre in Perth, while 5 of the ETS medical practitioner participants were based in Perth, 6 were based in regional Western Australia, 4 were overseas, and 1 was interstate. Two senior rural GPs provided their perspectives from both the receiving and provisioning ends of the ETS (as GPs working in regional locations). Around a quarter of ETS doctors and 16% of ETS nurses in the Command Centre participated in the interviews. All ETS nurses, except 1, were recruited by the same clinical researcher through face-to-face invitations, while 83% (15/18) of the ETS doctors who participated in the interviews responded to the initial invitation to participate. The purposive recruitment yielded 2 additional ETS participants to add weight to the perspectives of overseas or interstate doctors. Multimedia Appendix 2 lists the details of the participants, including home base, professional designation, gender, ETS role, and perspectives they contributed to this study.

### Domains That Emerged From the Simultaneous Data Collection and Analysis

**Figure 1:**
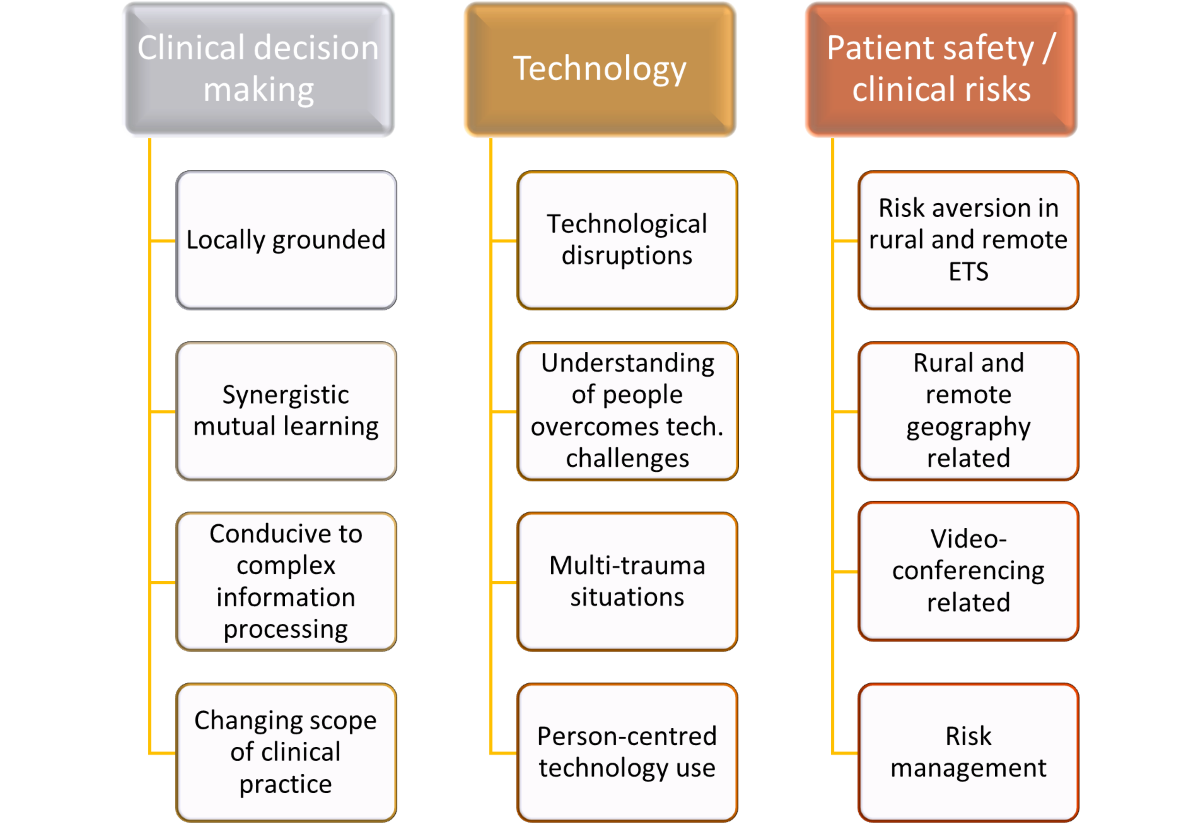
Thematic domains and subthemes that emerged in this study. ETS: Emergency Telehealth Service; Tech.: technology.

The major thematic domains identified in this analysis were clinical decision-making, technology, and patient safety or clinical risk. The themes and subthemes have been outlined below. Figure 1 provides a representation of the domains and themes.

### Clinical Decision-Making: Benefits of Involving the ETS to Support Clinical Decisions

Participants identified 3 key benefits of ETS involvement in rural and remote clinical services. Hospital clinicians reported that having the ETS improved the willingness and confidence of regional clinicians to practice in remote locations. The ETS enabled synergistic mutual learning through the collaborative patient management of site-based and ETS clinicians. Further, ETS FACEMs experienced the benefit of ETS practice over site-based practice as it allowed them to be more cognitively focused when processing complex information. Participants touched on the changing scope of clinical practice as part of their discourse on clinical decision support.

#### Locally Grounded Clinical Decisions

Participants described timely and appropriate disposition (admission, discharge, or transfer) of patients as the most significant clinical decision for rural and remote EDs. Disposition of ED patients, especially from less-resourced remote EDs, has significant patient safety and clinical risk implications. Clinical decisions pertaining to patients presenting to small and remote EDs differed from those of better-resourced urban or regional center EDs as site-related factors needed to be considered.

All participants commented that multiple interacting factors were at play when deciding to transfer a patient for ongoing management. Table 2 lists the factors they considered in their clinical reasoning, and only 1 was related to technology. The predominant consideration was the resources available locally within the presenting hospital and in the local community. The next consideration was the distance to a destination site of definitive treatment if a patient’s condition deteriorated. The final consideration was the timing of transfer and its appropriateness according to an individual patient’s health condition.

**Table 2:** Thought processes in clinical decisions including patient disposition considerations via rural and remote emergency telehealth.

Thought processes of decision-making clinicians	Technology related	Hospital/location related	Patient related	Emergency medicine related
Normal diagnostic decision-making: what is wrong with the patient?	No	No	No	Yes
Can the patient be adequately assessed for all relevant possibilities at the site? What [differential diagnosis] am I potentially missing and is that putting the patient at risk?	Yes	Yes	No	No
Is this patient safe to go home medically and socially?	No	Yes	Yes	No
Is there a local GP^a^ in town to care for the patient the next day?	No	Yes	No	No
Am I happy for the patient to wait till the next day for follow-up in a community?	No	Yes	Yes	Yes
Considering the patient’s location, the weather conditions, and the local resources available, is the patient safe to stay at this site and/or be admitted locally for observation?	No	Yes	No	No
Does the patient need to be retrieved earlier [compared to the standard of a tertiary center or regional center ED^b^]?	No	Yes	No	No
Where and how do I transfer them? Is this patient safe to go somewhere with private transport or road ambulance, or do they require aeromedical transfer?	No	Yes	Yes	No
What does the patient want? (some patients do not want to leave their community)	No	No	Yes	No

^a^GP: general practitioner.

^b^ED: emergency department.

#### Synergistic Mutual Learning With Joint Clinical Decision-Making

ETS physicians were reliant on the local knowledge of hospital clinicians at the site of patient presentation to facilitate and consider community, social, and resource factors in clinical decisions for regional patients. Rural-based hospital clinicians reported benefits from the assistance of ETS clinicians in making clinical decisions, especially their support in connecting to appropriate and available resources.

Regional-based participants reported that the involvement of ETS colleagues supported them in answering the questions listed in Table 2, with increased speed and confidence. An ETS physician who also worked at a site based on a WACHS ED stated:

It makes certain that we make the best decisions for our patients. ETS does make the decision-making process much easier in the region. I've got somebody else online who can call me if I'm looking after somebody who's critically ill. They can help organize transfers and ICU admission, they can help me with drug doses, so I can…crack on and do what I need to do. And I know that I'm working in a safe environment.

ETS Doctor #3

Participants also stated that they acquired a different skill set working in a supportive role to clinicians in remote facilities, including ways of communicating instructions to presenting hospital clinicians (mainly nursing staff), gathering medical history, prescribing medications, and efficiently analyzing and synthesizing presenting complaints.

For ETS clinicians who were FACEMs mainly based in metropolitan hospitals, participation in ETS consultations provided a new and unfiltered experience in the rural and remote clinical practice. Many reported that this changed the way they practiced in their substantive role on the floor of tertiary EDs:

I think [the learning has been] in both directions, one, it makes me realize how resource rich we are in a tertiary hospital. Two, it makes me realize that the nurses and other staff out there [in regional WA] are often just as competent, just as knowledgeable, and just as skillful and often braver than we are in tertiary hospitals. It certainly helps me stay grounded when I'm accepting referrals. [Tertiary hospital] has a big catchment, we receive a lot of these people from peripheral hospitals and nurse run clinics. And one that certainly helped me know where all these places are, when I'm on the receiving end of the phone…I'm able to explain to other staff exactly how resource limited, a lot of these clinics are. And there is a reason they haven't had blood tests and haven't had imaging. And in fact, there's no doctor there. So they have just been seen by nurses must come to be seen by us, we don't have to be difficult about accepting them. I think that's been quite useful.

ETS Doctor #43

#### Conducive to Complex Clinical Information Processing

Participants reported that their ability to control and prioritize their consultations in the virtual environment assisted their efficiency in processing complex information and enabled more objective assessment than working in person on the ED floor, given their distance from the patient. Participants reported the benefits of providing virtual care compared to working on an ED floor:

If you're on an ED floor you are interrupted constantly because you're physically there…one of the most frustrating things is you're constantly distracted, and you can't finish a task from A to B, I think that adds hugely to burnout. …But you are focused here [at ETS], …we can only see one camera…one patient at a time. …And the chaos is more manageable… you can choose to not be interrupted. So I feel that it's very satisfying, because you're so cognitively focused. And I think that can potentially lead to better quality care… because you can just make better decisions.

ETS Doctor #6

With the ability to control the environment they practice in, ETS FACEMs found the virtual environment conducive to them being more cognitively focused.

#### Changed Scope of Clinical Practice

The impacts on the scope of practice of regionally based clinicians, especially nurses, were dichotomously reported. Most participants reported an increasing scope of site-based clinical practice with ETS doctors guiding local nurses to perform procedures that they otherwise would not have the confidence to do on their own. A contrary perspective was the engagement of ETS clinicians in treatment and procedures or clinical decisions, which local clinicians would have performed independently through a local process in place prior to ETS implementation. For example, regarding giving a tetanus booster, 1 participant commented:

…we'll get consults of patients who need a tetanus booster, they've had a small wound a few days ago, …I don't know what they would have done before, I think they would have done it and had a process in place to do it.

ETS Doctor #2

The changing scope of practice was noted by the participants. This reflected policy changes to increase medical practitioner involvement in ED consultations and was an emerging theme that warrants further in-depth exploration.

### Technology

The second thematic domain was technology, which included technological (digital) disruption and mitigation strategies in emergency telehealth consultations.

Participants were of the view that the WACHS ETS had already made most of the technological and process improvements and considered that any further changes on the technological front would have incremental benefits in effectiveness for improving patient health outcomes. One FACEM participant stated:

A lot of the system problems are the fact that we have a hundred places that are tiny and it’s hard to get [for example] a pathologist, or experienced nurses for long stays. A lot of it is system problems that are difficult to modify.

ETS Doctor #43

We probably made the easy gains and the incremental changes you can make to improve things from here are big and costly, and difficult. For example, attracting and keeping people in remote communities and providing enough staffing to let GPs take holidays and do professional development and be replaced by someone, rather than leaving the community to rely on telehealth because there is no one else to cover.

ETS Doctor #43

Participants described the ways in which they modify how they use the telehealth technology or adjust the process of care to improve the quality of ETS consultations.

#### Technological (Digital) Disruptions

A commonly expressed view among ETS physician participants was that during videoconferencing, one is expected to lose nonvisual cues and some verbal cues due to technological (digital) disruptions. Clinicians reflecting on the videoconference consultation experience suggested that the amount and quality of information a clinician could gather through video came down to their level of understanding of people as individuals. They referred to the nuances of patients’ expressions observable during videoconferencing and being able to relate to the regional context of the patients. Table 3 provides a summary of the circumstances around the technological barriers during an ETS consultation and the mitigation strategies participants used to overcome these technological disruptions.

**Table 3:** Technology-related issues of Emergency Telehealth Service technology and mitigation strategies.

ETS^a^ technology-related issues	Technology barrier				Mitigation strategy		
	Barrier	Circumstances	Participants	Strategy		Incompatibility	Participants
Positioning of the monitor where the image of the ETS physician appears (ie, at the foot of the ED^b^ stretcher)	Sending the wrong message that the doctor is looking down on the patient. Abrupt appearance does not allow ETS to ease into the scene as in the case of an in-person ED floor.	When people on the floor are not adequately prepared and people are unaware why the ETS clinician is appearing on the screen, there can be disruptions in the actions already taking place in the ETS bay.	ETSD^c^ #1, 4, 6, 7, 10, and 13; ETSN^d^ #3	Prepare patients before the VC^e^ link is up; explain the process before and after VC consultation, including the steps leading up to transfer when interhospital transfer is indicated ETS physician is identified as not from Perth and thus does not appear as “someone from Perth talking down on us”		In a critical encounter, there is no time and space to prepare patients and people in the room. Only available to regional-based ETS clinicians.	ETSN #3; ETSD #7
Talking into an open space; broadcasting of sound from the VC setup into the ETS bay	Impersonal, with privacy and confidentiality potentially compromised; concurrent private conversations can occur from adjacent ED bays, which can create distracting noise during the consultation process.	Especially when multiple parties are involved in the conversation	ETSD #1, 4, 7, 10, and 13; ETSN #3	Mute VC sound and use a telephone directly with site-based clinicians or patients for audio to ensure private conversations. Transition to telephone only after an initial meet and greet with VC. Clinicians on the floor are encouraged to wear headphones to enable switching voice channels between different target audiences.		If children are involved, communicate with parents or carers	ETSN #3 and 4
The ETS clinician’s visual field of the ETS bay is restricted by the camera’s visual field	ETS clinicians are not aware of others in the room.	Consulting before realizing a family member or another patient is in the room; knowing who is in the room and their relationship with the patient can change the dialogue and alter the instructions given regarding the next steps.	ETSD #1 and 6	Use a telephone before screen appearance, find out who is present, and clarify the purpose of involvement. Self-introduction, role of ETS consultation, and a name shown at the base of the screen.		—^f^	ETSD #2, 4, 8, 9, 12, 13, 15, and 43
Zoom in or out function adjusted locally without the ETS clinician’s awareness	Unable to zoom in to see what needs to be seen.	Significant (disastrous) impact in critical situations.	ETSD #5	Having a reliable camera that the ETS doctor can control.		—	ETSD #4 and 14

^a^ETS: Emergency Telehealth Service.

^b^ED: emergency department.

^c^ETSD: ETS doctor.

^d^ETSN: ETS nurse.

^e^VC: videoconferencing.

^f^Not applicable.

The videoconferencing equipment setup and the restriction of the camera’s visual field created technological barriers affecting the quality of ETS consultation. Physical distance was the perceived distance between either side of the high-definition audio-visual equipment installed in the resuscitation bay of the ED at each site.

Participants experienced constraints of the camera setup made consultations impersonal and visual cues easily missed or distorted, and they shared how they adapted in their ETS practice to ensure effective visualization. One doctor said the technical issues and logistics of commencing a consultation with sudden appearance on a screen and sound broadcasting as key differences to working on the floor:

…, you got to spend time to dial in, and, then suddenly just appear on the screen in front of the patient… sometimes they're a bit shocked because you're big and loud on the screen… especially if they're drunk, confused that can exacerbate that [shock]. And sometimes I don't know what the volume is, sometimes I'm absolutely booming. And then everybody can hear me in the ED… they got to find the mute button, unmute me and decrease the volume…

ETS Doctor #6

A few participants suggested that adding a reliable remote-control pan or zoom would considerably improve the quality of the ETS physical assessment:

…great history and reading the cues if someone is lying very still, are they squirming around, are they making reasonable eye contact, are they coherent when they talk, all of that sort of stuff... you know looking for facial droop, obvious distal neurological deficit can be done really well [with reliable and high resolution camera].

ETS Doctor #14

#### Understanding of People Can Overcome Technological Challenges

Through reflective practice, participants redefined and modified their practice over the ETS to mitigate the shortcomings of the videoconference equipment setup. There were varied responses to technological (digital) disruptions in interpersonal communications. ETS doctors gave examples of how they used visual and imperfect auditory cues to assist with formulating their working diagnosis when direct eye contact was not possible via videoconferencing. Visual cues, such as closing hands and fidgeting, were used to the clinician’s advantage to help with diagnosis. Humor and a nonjudgmental approach were helpful in getting patients to reveal their alcohol issues and to work collaboratively with patients over videoconferencing to reach the best outcome for them. These human approaches made a notable difference in the virtual physical assessment process.

#### Multitrauma Situations With One Videoconference Bay

In a multitrauma situation when only 1 doctor was available on site, teamwork was required. For example, when the site-based doctor was busy with hands-on procedures in collaboration with an ETS FACEM, a local nurse stepped up to perform the team leader tasks under the guidance of ETS clinical nurses. When there were multiple casualties, all casualties would need to rotate to be in front of the cameras (typically 2 perpendicular pan/tilt/zoom cameras), and by using headphone channel switching, ETS clinicians could converse with each of the site clinicians individually. When there was a requirement to “shuffle” beds in and out of the camera visual field, adequate explanation ahead of the action was noted as an indispensable component of patient-clinician communication.

#### Person-Centered Technology Use

Facing variations in the quality of the internet connection, fixed positioning of cameras, and the broadcasting of sound into an open space of the ETS bay at regional EDs, ETS clinicians developed approaches to work with and via the presenting hospital clinicians to improve the quality of videoconference consultation while also protecting the privacy and confidentiality of patients. Overcoming technological limitations required users to troubleshoot and adapt to circumstances, requiring flexibility to ensure the effectiveness of virtual care. This finding pertains more to the exigent nature of EM and less to the rural and remote context of ETS delivery.

### Patient Safety and Clinical Risk Considerations

The final thematic domain was related to the perceived risks of virtual care identified by the study participants (Table 4). The central aim was to ensure patient safety when using telehealth. If the ETS team perceived that patient safety was compromised by remaining at the local facility (particularly if there was no on-site medical practitioner), they would strongly consider transferring patients to an appropriate hospital for ongoing management. ETS clinicians who had higher levels of perceived clinical risk had a lower threshold to transferring their patients. Participants were confident that with experience (both at the site and virtually), some transfers could be avoided. The modifiability of risks as assessed in this study identified areas for potential improvement and the factors to consider in the effectiveness dialogue.

**Table 4:** Factors that make emergency telemedicine clinicians risk averse in rural and remote areas in Western Australia.

Clinical risks and sources of risks in the Emergency Telehealth Service practice		Technology related	Hospital/location related	Emergency medicine related	Is it modifiable?
**Insufficient or lack of local resources**					
	Nature of ED^a^ cases: sudden deterioration not always predictable	No	No	Yes	Not modifiable
	Distance/time required to transfer to a location with more resources against the risk of deterioration	No	Yes	No	Not modifiable
	No short-stay unit: cannot keep the patient after hours	No	Yes	No	Potentially
**Local workforce capacity and capability**					
	Clinicians on the ED floor are unable to perform the required physical assessment	No	Yes	No	Limited
	Procedural (IV^b^ access and treatment procedures)	No	Yes	No	Potentially
	No doctor or insufficient nurses onsite to admit the patient	No	Yes	No	Potentially
	No GP^c^ in town to follow-up on the next day	No	Yes	No	Limited
**Unable to secure a clear diagnosis**					
	Unable to access required testing or imaging	No	Yes	No	Not modifiable
	Unable to fully examine: chances of missing a clinical cue are greater	Yes	No	No	Limited
	Not 100% confident with the clinical picture despite full complement of clinical information	No	No	Yes	Not modifiable
	Variable internet quality: disrupts physical assessment	Yes	No	No	Limited
	Camera is not always reliable	Yes	No	No	Modifiable
**Individual factors**					
	Physician personality and approaches to risk	No	No	Yes	Not modifiable
	Physicians are less comfortable with the reliability of their own physical assessment over VC^d^ compared to face-to-face evaluation	Yes	No	No	Potentially
	Patient preference (to stay closer to home)	No	No	Yes	Not modifiable
	Patients and family members are risk averse to telehealth consultation	Yes	No	No	Potentially

^a^ED: emergency department.

^b^IV: intravenous.

^c^GP: general practitioner.

^d^VC: videoconferencing.

#### Concept of Risk Aversion in the WACHS ETS

Risk aversion impacts transfer rates from the site of presentation. The level of risk ETS clinicians were willing to take in keeping patients locally depended on 4 broad categories risk considerations. The first and second considerations of risks were related to the presenting hospital capacity and capability (ie, the health service resources and workforce). Risk aversion was associated with increasing remoteness of the presenting hospital. The third category of risk was unanimously reported by all the ETS physician participants. They reported that they were not able to examine the patient adequately or be completely confident with the clinical information relayed by site-based clinicians. This concern reflected how well ETS clinicians felt they could apply strategies to mitigate technological (digital) disruptions. The final category reflected individual clinician factors, such as their personality and their own tendency to be risk averse, and was present in EM generally, irrespective of whether it was place based or virtual. Factors related to the nature of EM and patients presenting to EDs would not have changed this risk even if managing clinicians were able to be “teleported” to the scene in person, and these aspects have not been discussed further in this paper.

#### Risk Aversion Associated With the Hospital of Presentation and the Rural and Remote Geography

Of 17 risks, 8 emerged from the ETS risk-aversion discussion related to the hospital of presentation and the rural and remote geography. These were less readily modifiable than the videoconference-related risks. The least modifiable risk was related to the inability to access the required tests or imaging, and this was reported by multiple clinicians. If point-of-care testing and the pathological approach available locally were not adequate to reach a clear diagnosis, the patient would be transferred for further investigation. The extent to which this was considered an issue varied, with 1 participant (ETS doctor #3) having a view that point-of-care tests, routine pathology, and x-ray imaging available locally were generally satisfactory and sufficient for many of the presentations, and this view differed from that of many other participants.

Another factor associated with the presenting health facility was related to the regional workforce capacity and capability to perform the required physical assessment or procedure, or to keep patients locally for observation onsite or in the community. This mainly applied to sites where there were no medical practitioners. These factors had limited modifiability at the time of the consultation but were potentially adaptable. When the ETS clinician was unable to work with the clinician onsite to overcome the limitations of physical assessment over videoconferencing, the workforce capacity at the presenting hospital impacted the ability to secure a clear diagnosis and manage the patient adequately. Workforce capability was also related to the inability to perform required procedures ranging from establishing vascular access, inserting an intraosseous needle, and simple suturing to performing more complicated procedures, which could have avoided transfer. Generally, this is related to nursing-only facilities staffed by clinicians with limited scope of practice. Even when a diagnosis was made and the required procedures are performed, a patient might still be transferred if there was no doctor or insufficient nursing capacity to keep the patient on site for further observation, including if there was no GP in town the next day to follow-up in the community.

The final factor adding to the risk aversion of ETS clinicians was related to resources available at the hospital of presentation. The key issue raised here was the absence of a short stay–type inpatient unit to keep patients in the hospital for overnight observation. The distance and time required to transfer patients to a location with higher levels of capacity and capability were considered against the risk of deterioration of the patient’s condition and the assessment of clinical risk. These factors were not modifiable and subject to the treating clinician’s judgment of the patient’s health condition.

#### Videoconference-Related Risk Aversion

Of the 4 categories of ETS clinical risk, 2 included factors that were related to the inability to secure a clear diagnosis and were related to videoconferencing. Many participants discussed the need to transfer patients because they were not confident with the clinical assessment via video. These included their perception of missing significant clinical signs, due to being unable to directly palpate or examine the patient, which impeded reaching a clear diagnosis. For example, 1 FACEM participant commented as follows:

…spending a lot more time thinking about exactly what I’m going to do and why… you don’t have any of the nice reassurance normal investigations you would normally have. You are putting yourself more at risk by doing telemedicine because the chances of missing something are greater because you don’t have access to all the little things that would make you much more comfortable.

ETS Doctor #14

ETS physician participants also reported issues with internet connectivity and speed, and many made comments on issues associated with the reliability of the camera affecting their ability to formulate a clear diagnosis. As discussed above, issues related to the camera were associated with the way it was physically positioned in the allocated treatment bay; operational limitations with pan, tilt, or zoom; and the camera technology itself. The internet speed and connectivity were more challenging as they were impacted by the internet provision where both the patients and ETS clinicians were physically based (nationally and internationally).

Finally, user acceptance of videoconference consultation differed among participants. Not seeing patients face-to-face made some clinicians more cautious and risk averse, with treating clinicians also reporting that some patients or family members were risk averse to telehealth consultations due to a lack of experience or understanding of this alternative modality of clinical service delivery. Several ETS clinicians reported that they spent more time second-guessing their observations and decisions, suspecting that they are less reliable over videoconferencing compared to a face-to-face meeting.

#### Risk Management for Patient Safety Instead of Risk Aversion

Complementing the higher level of potential and perceived risk associated with videoconference consultations, participants shared a range of perspectives on the perception of clinical risk:

…am I risk averse? I am probably less. It's not my personality. My personality is not risk averse. And I think most emergency physicians are not risk averse. That's not the nature of our business. There's risk everywhere we are risk managers.

ETS Doctor #2

Despite observing the potential risks associated with videoconference consultations, participants believed that those who were overly risk averse would not participate in telehealth, and it was a matter of finding a balance between the perceived benefits of ETS and the expected risks associated. A pragmatic view was that ETS physicians were no more cautious on camera compared to being on the ED floor; however, due to incomplete data points to inform an evidence-based clinical decision, interhospital transfer may be suggested for the required investigations. In terms of confidence in their decisions, some participants reported that they felt comfortable with their clinical decision-making as FACEM training helped balance relatively scant information in undifferentiated patients, and this was no different from the decision they would make in a face-to-face meeting:

Well, that's my job. It’s not so much about being confident. Well, you've got to be confident in your decision because otherwise you can't afford to be in the [emergency medicine] business. I mean, you know, if you if you're worried about the patient, then you do something to alleviate that worry… Occasionally, there are times when it is difficult… for example, if a patient has a communication problem in either they are deaf, or they can't speak properly, and so my interaction is sufficiently degraded but otherwise, for [most] times, I don't think it does make a particular difference to be honest. The difference between me not being there and being there is far more evident in the skills that are brought face-to-face. And those are nearly all procedurals.

ETS Doctor #5

## Discussion

### Overview

The experiences and perspectives of WACHS ETS clinicians contributed to an in-depth understanding of the factors influencing clinical decision-making during an emergency telehealth consultation in rural and remote settings. The study progressed the understanding of the mutual learning opportunities presented during the joint decision-making process, the perceived clinical risks of emergency telehealth practice in the rural and remote context, and the technological barriers imposed on clinical decisions and the modifiability of these perceived risks.

### Principal Results: Meeting the Principles of the ETS Practice

This research described the mutually beneficial effects of ETS consultation recognized by the participants in their clinical practice. These were in line with the principles and interim standards stipulated by the Colleges of Emergency Medicine that telehealth should complement and be part of the regional model of emergency care delivery validated by all stakeholders [1-3]. This finding also aligns with previous research, which highlights how rural and remote ED clinicians rely on the support and expertise of their colleagues to navigate and succeed in the unique challenges of rural and remote settings [8-10,17,18]. It further explored how this dynamic operated when colleagues provided support through a virtual presence. This research provides additional insights into how collegiality during emergency telehealth consultations supports various aspects of practice. These include enhancing confidence in rural and remote clinical practices, advancing the practice of virtual EM, and incorporating the rural and remote context into collaborative clinical decision-making involving stakeholders on both sides of the technology.

### Clinical Decision-Making

The participants described clinical decision-making mediated by a complement of ordinary technologies. Considerable regard was given to the context of the hospital of initial ED presentation, the community where the patient is from, and the regional Western Australia health service delivery landscape. ETS clinicians were reliant on the local knowledge of presenting hospital clinicians, the patients, and their family members to incorporate these local contexts into collaborative decision-making. The capacity building was mutually beneficial for regionally based clinicians and ETS clinicians. Their complementary skills and synergies in what they were able to contribute to patient care became apparent during ETS consultations. In this paper, we have referred to this phenomenon as “synergistically mutual learning.”

Many participants described their experience of improved efficiency in processing complex clinical information through better focus. This reflected the time-sensitive nature of the ED, especially when the hospital of definitive treatment was hours away, and the organized chaos in multitrauma situations. This finding represents a novel contribution from the perspective of emergency physicians conducting virtual ED consultations. The quality of clinical reasoning was reported to be superior in ETS practice where there was greater reliance on synthesizing medical history, reviewing clinical records, and carrying out focused processing of multiple sources of information. In situations where there was a heavy reliance on physical examinations and complex laboratory or imaging investigations, clinicians were cognizant of the peculiarities and distinctive requirements of the specific conditions and replicated, as much as possible, the components of an EM consultation. This was in line with the guiding principles stipulated by the Colleges of Emergency Medicine to deliver a service of similar quality and standards as ordinary care [1,2].

The perspectives of ETS clinicians highlight a critical aspect of improvement in the overall efficiency of emergency care delivery not previously reported in studies of the efficiency of emergency telemedicine within receiving hospitals [10-12,14-16].

### Clinical Use of Technology

At the forefront of technological change is how humans personalize technologies. The way technologies are used is a significant part of the innovation in the EM practice in rural and remote settings. This study extended past findings of technological difficulties impeding care processes [10,14,16,19-21,24-26], patient privacy, confidentiality, and data security [24-26] by explaining how emergency telehealth practitioners introduced human factors to the innovative use of emergency telehealth through their understanding of people and tailoring the use of the same technology to the needs of patients on the other side of the camera as described in Table 3. To the best of our knowledge, this is the first description of how human users innovate to close the distance across technological barriers in an emergency telehealth scenario.

### Modifiability of Patient Safety and Clinical Risks Identified

Risk factors related to ETS technology were modified through upgraded camera resolution, fixed camera positioning, increased user experience, improvisation, and consumer preparation. The quality of physical examinations was generally considered unlikely to match the quality of face-to-face examinations, especially when examinations of hard-to-visualize areas of the body or procedural interventions were indicated. However, emergency telemedicine using videoconference facilities has been considered to have reached a plateau and might be sufficient to cover a great majority of presentations. Additional investment in other examination equipment will need to be carefully evaluated as the benefits of further equipment upgrades are likely to be incremental against the costs of purchasing the equipment.

To meet the standard of replicating components of an EM consultation stipulated in the interim guidance [1,2], the need for an appropriately skilled and competent local workforce cannot be overlooked. Workforce capabilities were potentially modifiable through education, including pre- and postvocational training opportunities; however, modifiability was offset by the ongoing issue of the turnover of clinical staff in rural and remote hospitals. This said, participants provided examples of how inter- and intraregional movement of clinical staff helped to retain the necessary skill sets within Western Australia. The extent to which clinical skills acquired through ETS involvement in ED consultations benefited the overall capability of the regional clinical workforce in WA fell outside the scope of this paper; however, this is an area of significant importance and relevance to the effectiveness of the ETS.

The pivotal but nonmodifiable consideration in clinical decision-making was the physical distance from definitive treatment locations and presenting hospitals. Local hospital resources, capacity and capability were beyond the scope of this study, but it was acknowledged that these had limited modifiability, at least in the short term.

This paper did not explore the impact of the ETS on the quality and standard of care in larger regional EDs, and the dominant view that EM specialists are risk managers is of significance. Any manifested risk aversion is likely related to patient safety considerations given that rural and remote contextual factors are nonmodifiable or have limited modifiability. Despite the dominant view that the ETS added value to presenting hospital clinical decisions, there were also concerns that pre-existing processes and capacity had been disrupted by the care processes now available through the ETS. The change in the scope of practice in presenting hospitals, particularly regarding nursing staff, because of ETS involvement is a subject for further detailed exploration to reach an optimal balance.

### Novel Contributions of This Study

Prior work has paid considerable attention to advocating for telehealth policy reform [30] and identifying the barriers and enablers of telehealth implementation in rural and remote EDs. This is the first study to systematically detail the complex interrelationships between technological and human interfaces. The barriers to quality and safety of emergency telehealth consultations and mitigation strategies used by the WACHS ETS clinicians when collaborating with rural and remote EDs were detailed. These perceived clinical risks and risk mitigation strategies added new insights into the contextual factors influencing the effective implementation of rural and remote ETS.

Findings around technological challenges were consistent with the findings of previous studies that pointed to variations in usability, depending on the setting and situation. Unlike previous studies that compared the approach of different types or combinations of telehealth technologies (eg, videoconferencing, store and forward, and telemonitoring) [21], this study embraced the use of available technology as a norm in rural and remote ETS practices in Western Australia. The focus on the strategies used in WACHS ETS delivery to overcome technological barriers in this study makes a novel contribution to advancing our knowledge on the modifiability of the risks associated with rural and remote virtual care and helps to inform future quantitative analysis and interpretation of findings on the effectiveness of rural and remote ETS.

### Limitations and Future Research

The findings reported in this paper are subject to several limitations. Clinical nurse coordinators are the first contact points for accessing the ETS, and this triaging (referred to as ETS prioritization to distinguish from the Australasian Triage Scoring system) plays a pivotal role in case review order and selection. The ETS prioritization process ensured that the presenting hospital and community context were incorporated into timely and appropriate care from the first point of ETS contact. This paper focused on regional context considerations influencing clinical decisions around the disposition of ED patients during a virtual consultation and did not examine regional context factors already incorporated during the process of ETS prioritization. Future reports on ETS prioritization would be an essential addition to the body of knowledge on emergency telehealth implementation in the rural and remote context.

Telehealth, especially emergency telehealth, is a relatively new area in clinical practice with evolving clinical governance standards. Medical indemnity issues were discussed during the interviews but did not emerge as a dominant theme in the data analysis. WACHS ETS physicians are particularly focused on patient safety. However, based on available data, it is difficult to conclude the extent to which medicolegal considerations are at play in the response to the risks associated with the ETS practice.

The financial viability or cost-effectiveness of virtual care is best explored quantitatively. It is worth noting that participants were value conscious in evaluating disposition options. However, they were not able to comment on the financial viability of the service as it was not within the role of most of these clinician participants. The implementation factors identified inevitably influence the effectiveness of telehealth in rural and remote EDs but are insufficient to determine the effectiveness of the ETS. This study represents a step toward determining the effectiveness of ETS delivery and is an area for future research.

The recruitment of participants may have been subject to social desirability bias, and some nonresponders may have had an alternative opinion about the service, which they felt did not align with the health service’s official position. The presenting health condition is one of the central tenets in this research, although the data collection did not focus on specific health conditions. Our intention in this paper was to capture the overarching themes of clinical decision-making and how they affect the safe and effective delivery of rural and remote ETS to improve patient health outcomes. There were no comparisons between the treatments of urgent and nonurgent presentations because formative analysis showed the tendency of using the ETS for more urgent cases. This focus was due to the higher proportion of FACEMs among the study participants, who were more likely to encounter urgent cases.

### Conclusion

Telehealth has the potential to improve the processes of clinical decision-making in rural and remote EDs. The participants, all of whom participated in delivering the ETS, perceived improved accuracy, confidence, and speed of clinical decisions. The reach and effectiveness of the ETS in addressing the identified health needs remain to be tested.

Patient safety was central to all clinical decisions, which were grounded in the capability of presenting hospitals and the local community context. The risks of delay in definitive care for those patients requiring transfer for ongoing management associated with the geographical distance to the hospital for definitive treatment and the presenting hospital capability were not amenable to change. The importance of social and geographical contexts for appropriate telehealth delivery was highlighted in this study.

Technological barriers to the safe and effective delivery of the ETS were amenable to improvisation and improvements to infrastructure, but overall, participants were aware that they could not reach the same reliability as that in face-to-face consultation for all presenting conditions. Although the ETS was an acceptable and feasible option for ED consultation, the maintenance and sustainability of the ETS as part of routine practice and policy are dependent on presenting conditions.

Investment into rural and remote telehealth has the potential to support regionally based clinicians both directly in patient care and indirectly by promoting their (health workforce) longevity in regional areas through the ongoing development of the virtual care network. The effect of the ETS on the scope of the regional ED practice and the building of clinical skills within the region warrants further in-depth exploration including its potential implications for the overall effectiveness and cost-effectiveness of telehealth services in rural and remote EDs.

## Appendix

Multimedia Appendix 1 Interview guide for Emergency Telehealth Service clinicians.

Multimedia Appendix 2 The professional designations and perspectives of study participants.

## Abbreviations

ACEM: Australasian College of Emergency Medicine
ANZFTCOP: Australian New Zealand FACEM Telemedicine Community of Practice
ED: emergency department
EM: emergency medicine
ETS: Emergency Telehealth Service
FACEM: Fellow of Australasian College of Emergency Medicine
GP: general practitioner
WACHS: WA Country Health Service
