# Supplementary material for: Perceived Risks, Mitigation Strategies, and Modifiability of Telehealth in Rural and Remote Emergency Departments: Qualitative Exploration Study
Source: JMIR Hum Factors. 2025 Apr 15;12:e58851. doi: 10.2196/58851 (PMC12041817; doi:10.2196/58851)
Supplement: Multimedia Appendix 1 [file humanfactors_v12i1e58851_app1.docx]

**Multimedia Appendix 1.** Interview guide for Emergency Telehealth Service clinicians.

| **Area 1: ETS Model of Care** | **Area 2: Workforce Support Model** | **Area 3: Clinical Decision Making** |
| --- | --- | --- |
| Explore health service providers views and perspectives on current WACHS ETS provision and models of care. | Elicit remote clinicians’ experience after critical or difficult encounters via telehealth e.g. failed resuscitation, crisis resource management, communication difficulties, and adverse outcomes, and explore what formal and informal support have been accessed. | Explore the concept of ‘risk-averse’ in the context of ETS delivery and the impact this can have on clinical decision making. |
| **Question preamble**  The workforce ‘parties’ involved in an ETS consult include the ETS physician, local doctors (usually GPs), nurses at the Command Centre, local nurses. Each member of the team plays a different role in an ETS consultation and has different workforce support needs during and after ETS encounters. *NOTE 1 | **Question preamble**  The geographic and time zone separation among ETS clinical teams creates unique circumstances around how, from whom and where clinicians debrief and support after critical and challenging encounters.  This question will also elicit clinicians’ perspectives on the effectiveness of current models of workforce support and explore ideas and other strategies that may be worthwhile pursuing to ensure the wellbeing of the ETS workforce. | **Question preamble**  Medicine has become risk-averse in general because of the fear of legal challenges against doctors’ medical licenses. Physical examination via videoconference is different from face-to-face and dependent on the accurate capture of verbal and non-verbal cues and physical examination capability of local clinicians. This can result in a lower threshold of recommending a transfer i.e. more risk averse. |
| **Main question:**  We are interested in your experiences working with the ETS – what role do you play in an ETS consult and how does your role function in collaboration with other members of the multidisciplinary team? | **Main question:**  What support does ETS offer to your work in the ED?  What strategy (strategies) have you used to debrief after a critical or challenging encounter?  What strategies do you use in and out of business hours?  Does this differ during COVID-19 lock down? | **Main question:**  What are the three most important clinical decisions you must make as an ETS physician?  How does telehealth affect clinical decisions? E.g. would you treat a person with abdominal pain the same way using ETS as a face-to-face consult?  What strategies can be put in place to improve confidence in clinical decision making? |
| **ETS physician**  Where do you currently work when consulting for ETS?  Where do you prefer to work while on duty for ETS?  Talk me through the experience of taking ETS calls from home compared to being in the command centre.  How is consulting via telehealth different from seeing patients face-to-face? | **ETS physician probes**  Can you recall a critical encounter such as resuscitation or major trauma when you are involved via ETS when you require to seek support from other colleagues? What happened and how did you look for support during and after the event?  Do you feel supported during and after critical encounters?  What strategies could ETS put in place to improve its workforce support model? | **ETS physician probes**  How confident do you feel about the clinical decisions you make over telehealth compared to seeing patients face-to-face?  What influences clinical decision making in each setting?  Are these factors modifiable?  (e.g. local clinician capabilities, digital technology that may help to improve confidence of clinical decisions) |
| **Command centre nursing staff probes**  How is teamwork different via telehealth compared to working with your team in the same location? | **Command Centre nurse probes**  How does having an ETS physician physically located at the Command Centre add to the operation of the ETS?  Can you recall a critical encounter such as resuscitation or major trauma when you are involved via ETS when you require to seek support from other colleagues? What happened and how did you look for support during and after the event?  Do you feel supported during and after critical encounters?  What strategies could ETS put in place to improve its workforce support model? | **Command Centre nurse probes** |
| **Local doctor probes**  Talk me through the role ETS physicians and nurses based at the command centre play in clinical work at your ED. | **Local doctor probes / Local nurse probes**  How do you support each other as a team during and after critical encounters inclusive of local team and the ETS team?  Do you feel supported through critical encounters?  What strategies could ETS put in place to improve its workforce support model? | **Local doctor probes**  How has working with ETS physicians influenced your clinical decisions? |
| **Local nurse probes**  Talk me through the role ETS physicians and nurses plays in patient care at your ED. |  | **Local nurse probes**  How has working with ETS physicians change the way you care for patients in the ED? |

Final comments on teamwork work across different geographical locations and time zones and other strategies you have not already mentioned in the above to ensure workforce health and wellbeing.

Comments on care for patients presenting with stroke or stroke like symptoms.

Note 1: ETS encounters may be critical sick patients, crisis resource management due to resource exhaustion, or difficult communication encounters.
