# Supplementary material for: Perceived Risks, Mitigation Strategies, and Modifiability of Telehealth in Rural and Remote Emergency Departments: Qualitative Exploration Study
Source: JMIR Hum Factors. 2025 Apr 15;12:e58851. doi: 10.2196/58851 (PMC12041817; doi:10.2196/58851)
Supplement: Multimedia Appendix 2 [file humanfactors_v12i1e58851_app2.docx]

**Multimedia Appendix 2**. The professional designations and perspectives of study participants.

| **Participant ID** | **Homebase Category** | **Dr / Nurse** | **Gender** | **ETS Role** | **Perspective Category** | **Count** |
| --- | --- | --- | --- | --- | --- | --- |
| ETSN2; ETSN3; ETSN4; ETSN5 | Perth | Nurse | 3xF; 1xM | CNC | Provider | 4 |
| ETSD1; ETSD10  ETSD43 | Perth | Doctor | F | FACEM | Provider | 3 |
| ETSN1 | Perth | Nurse | F | Nurse educator | Provider | 1 |
| ETSD8 | Perth | Doctor | M | ED Registrar | Provider | 1 |
| ETSD15 | Overseas | Doctor | F | ED Registrar | Provider | 1 |
| ETSD6 | Interstate | Doctor | F | FACEM | Provider | 1 |
| ETSD5 | Overseas | Doctor | M | FACEM | Provider | 1 |
| ETSD14 | Overseas | Doctor | F | FACEM | Provider | 1 |
| ETSD11 | Perth | Doctor | M | FACEM | Provider | 1 |
| ETSD2 | Region | Doctor | M | FACEM | Provider | 1 |
| ETSD12 | Region | Doctor | F | FACEM | Provider | 1 |
| ETSD3 | Region | Doctor | M | GP | Both users and provider | 1 |
| ETSD4 | Region | Doctor | F | GP | Both users and provider | 1 |
| ETSD7 | Region | Doctor | M | GP | Provider | 1 |
| ETSD9 | Region | Doctor | F | GP | Provider | 1 |
| ETSD13 | Overseas | Doctor | F | GP | Provider | 1 |

Abbreviations: F = female; M = male; FACEM = Fellow of Australian College of Emergency Medicine; GP = General Practitioner; ED = emergency department; CNC = Clinical Nurse Coordinator; ETSN = ETS nurse; ETSD = ETS doctor.
